# A new tiny-leaved species of *Raveniopsis* (Rutaceae) from the Pakaraima Mountains of Guyana

**DOI:** 10.3897/phytokeys.91.14763

**Published:** 2017-11-28

**Authors:** Kenneth J. Wurdack

**Affiliations:** 1 Department of Botany, MRC-166, National Museum of Natural History, Smithsonian Institution, P.O. Box 37012, Washington, DC 20013-7012, USA

**Keywords:** Guiana Shield, Guyana, Kamakusa Mtn., leaf anatomy, *Raveniopsis*, Rutaceae, trichomes

## Abstract

*Raveniopsis microphyllus* K.Wurdack, **sp. nov.**, a new species known only from a single peak in the Pakaraima Mtns. of Guyana, is described and illustrated. This white-flowered shrub adds to the many narrow-endemic Guiana Shield species in the genus, and is unique in bearing small, trifoliate, sclerophyllous leaves. Leaf anatomy and surface micromorphology of the new species were examined to document its montane adaptations. The multiple trichome types of the leaves and flowers of new species were characterized, and the systematics value of the considerable foliar trichome variation in *Raveniopsis* is discussed.

## Introduction

The Rutaceae of the Guiana Shield region of northern South America contains about 60 species (nearly half endemic) in 20 genera (Kallunki 2005). Of those genera, three (*Apocaulon* R.S. Cowan, *Decagonocarpus* Engl., *Rutaneblina* Steyerm. & Luteyn) are small endemic groups and a fourth, *Raveniopsis* Gleason, is near endemic. *Raveniopsis*, the focus of this study, appears to be a classic Guiana Shield radiation where of its 19 currently recognized species, 16 are narrow montane endemics known from one or a few sandstone mountains (i.e., 13 are known from a single tepui including four species endemic to Sierra de la Neblina and three to Auyán-tepui) in Venezuela (Amazonas, Bolívar), Brazil (northern Amazonas), and Guyana (Mazaruni-Potaro, described herein). In addition, one species is more widespread and extends to lower elevations across this region, and two species are low-elevation disjuncts further south in Brazil (southeastern Amazonas, Rondônia).

*Raveniopsis* are attractive shrubs, mostly less than 2 m tall (up to 5 m in *R. stelligera* [R.S. Cowan] R.S. Cowan), with a diversity of leaf, trichome, and floral morphologies. Their leaves can be simple or trifoliate, and can be thin or clearly adapted for montane life in being thickened and well-covered in diverse types of trichomes. Among the species are two strikingly different floral syndromes that presumably reflect pollinator differences, and include: (1) short-tubed white to sometimes pinkish-tinged, or (2) longer-tubed red to orange corollas. The genus is characterized by a combination of pentamerous flowers, subequal and mostly free sepals that are longer than wide, androecia of two fertile anthers with basal appendages and three staminodes, zygomorphic tubular corollas with five lobes, and 5-parted apocarpous gynoecia producing follicular fruit.

Many of those diagnostic features also serve to group *Raveniopsis* with the “Angostura Alliance” (Galipeeae subtribe Galipeinae), which includes about 25 genera and 130 species restricted to the neotropics (Kubitzki et al. 2011; Bruniera et al. 2015). Many of the genera are small (13 have 1–2 species), and *Raveniopsis* is the second largest genus after *Conchocarpus* J.C. Mikan (53 species, including *Almeidea* St.-Hil.; Bruniera et al. 2015). Molecular phylogenetic studies have so far indicated that most of the limited Angostura Alliance taxon sampling (10 genera) form a core clade, and that taxonomic adjustments are needed to improve subtribal and generic circumscriptions (Groppo et al. 2008, 2012; Bruniera et al. 2015). Based on floral characters, *Raveniopsis* appears to be closely related to *Ravenia* and a further suggestion has been made that perhaps both genera should be combined (Kubitzki et al. 2011). *Raveniopsis* has not been sampled for any published molecular phylogenetic study, although preliminary evidence indicates that the genera should be kept separate (K. Wurdack unpublished).

While new exploration of montane areas of the Guiana Shield is expected to yield novelties, it was surprising that a very distinct new species of *Raveniopsis* was among collections made during recent botanical exploration of Kamakusa Mtn. in Guyana. The significance of this discovery was not known at the time of collection, although its identity was puzzled over by expedition members including the author. Kamakusa Mtn. is the highpoint of the Merume Mtns., a subrange within the Pakaraima Mtns., and forms part of an escarpment bordering the lowland (<100 m) rainforests of the lower Mazaruni River. This escarpment defines the eastern edge of a broad upland region (mostly >1000 m) within the Guiana Shield that continues westward into adjacent Venezuela and there includes part of the Gran Sabana and numerous scattered tepuis. While the summit of Kamakusa Mtn. apparently had not been botanically explored prior to 2012, an expedition in June–July 1960 lead by Stephen Tillett on behalf of The New York Botanical Garden traversed its lower slopes and made many type collections of taxa in diverse families (Wurdack et al. 2013). This richness in novelties suggests a higher degree of local endemism than observed in other mountains in the vicinity except Mt. Ayanganna, although many of those taxa remain poorly collected or studied.

## Material and methods

Scanning electron microscopy (SEM) was with a Zeiss EVO MA15 SEM at 10–12 kV after directly mounting dried herbarium specimen fragments, and sputter coating the samples with 25 nm of Au/Pd. For leaf anatomy, a rehydrated fragment was paraffin-embedded, sectioned at 10 μm, stained with toluidine blue O, and imaged with a Zeiss Universal Compound Microscope. The pollen was only examined and measured with SEM due to the few grains available. Trichome morphology and terminology follows Webster et al. (1996) that treats the great diversity of indument types in *Croton* L. (Euphorbiaceae), which like *Raveniopsis* has considerable variation in branched forms.

## Taxonomic treatment

### Raveniopsis microphyllus

Taxon classificationPlantaeSapindalesRutaceae

K.Wurdack sp. nov.

urn:lsid:ipni.org:names:77173688-1

Figure 1

#### Diagnosis.

Differs from *Raveniopsis breweri* in small trifoliate leaves, indument of small rosulate trichomes, subsessile and few-flowered inflorescences, smaller sepals and corollas, and hirsute anthers.

#### Type.

GUYANA. Cuyuni-Mazaruni Region: Summit of Kamakusa Mtn. (i.e., on top of 4th escarpment of four), impenetrable elfin forest to 3 m, extremely dense and wet, rich in epiphytes, 5°52'51.7"N, 60°6'10.4"W, 1686 m, 7 Jun 2012 (fl), *E. Tripp 3191* with K. Wurdack, A. Radosavljevic, and J. Ralph (holotype: BRG; isotypes: NY, US-3679224).

#### Description.

*Shrub* to 1.5 m, evergreen; leafy ultimate branchlets thin, 0.7–0.9 mm dia., densely pubescent; trichomes rosulate, 0.2 mm wide, sessile to shortly stipitate, with numerous short, uniform-length radii (arms), radii free without coherent edges; bark dark-brown, thin, easily peeled; bark of older twigs with fissures from periderm development, pubescence persistent on strips of remaining epidermis. *Stipules* absent. *Leaves* opposite, 3-foliolate, petiolate; petiole terete, 2.5–3 × 0.7 mm; leaflets elliptic, subequal, laminar size class leptophyll, margin entire, unlobed; terminal leaflet with petiolule 1–1.5 mm long, lamina 5.5–7.5 × 3–5.3 mm, length:width ratio 1.32–1.83 (mean=1.63, n=11), base cuneate, symmetric, apex angle acute, apex shape sub-acute to obtuse; lateral leaflets usually slightly larger than terminal, petiolules 0.3–0.5 mm long, lamina 5–8.8 × 3–4.5 mm, length:width ratio 1.67–1.97 (mean = 1.84, n = 11; measurements of lateral leaflets from same leaves used in prior terminal leaflet ratio), base subcordate, basal extension asymmetrical, proximal basal extension (outer lobe) 0.3 mm, distal basal extension (inner lobe) 0.1 mm, apex similar to terminal leaflet; adaxial side dark green in life, moderately pubescent, becoming glabrescent with age; adaxial trichomes multiradiate, radii 10–13, free, lateral radii 0.1–0.2 mm long, central radius sometimes differentiated by elongation to 0.6 mm and porrect (porrect-multiradiate), trichomes near leaf base and margins having the greatest central radius elongation; abaxial side densely pubescent with rosulate trichomes; blade coriaceous, pellucid dots not visible, cross-sectional profile 0.7 mm high including 0.4 mm lamina thickness plus 0.3 mm layer of abaxial trichomes, primary venation pinnate. *Inflorescence* terminal, subsessile, flowers 1(–3) in a reduced monochasium. *Flowers* bisexual, 5-merous, shortly pedicellate, pedicel to 1 mm long. *Calyx* 5-parted, sepals connate at base to 1mm, separate distally, coriaceous; sepal lobes erect, unequal, in 2 alternating size classes; longer 2, 2–3 × 1 mm, unequal with longest subtending banner petal lobe; shorter 3, 1–2 × 0.5 mm, subequal; externally pubescent with trichomes of both porrect-multiradiate and multiradiate types, internally glabrous and lined with files of dark-content cells. *Corolla* white, tubular-infundabuliform, of 5 connate petals, markedly zygomorphic, 12–15 mm long; tube 8–9 mm long, slightly curved, narrowest point at base 0.9–1.2 mm dia., distally expanded to 1.5–2 mm dia.; lobes 5, imbricate in bud, spreading at anthesis with 4 in 1 plane forming slightly recurved lip and 1 upright banner, 2–3.5 × 1.5–2 mm, subequal, internal pilose band below lobes (zone where androecium is similarly pilose), glabrous elsewhere internally and externally where imbricate petal margins overlap in bud, externally tube otherwise densely multiradiate pubescent becoming porrect-multiradiate towards lobes. *Androecium* of 2 fertile stamens and 3 staminodes, free from corolla. *Fertile-stamens* proximally fused, distally separate but coherent with tangled trichomes, 11.5–12 mm long; filaments 9–10 mm long × ca 0.4 mm wide (per filament) at base, expanding to 0.8 mm wide distally due to asymmetric wings (wider on outer edge, narrower on coherent edge), narrowly oblanceolate, flattened, <0.1 mm thick, central vein prominent; apex of filament abruptly narrowed as 0.3 × 0.1 mm extension connected to anther; fused base and apical extension glabrous, free part pubescent with long crinkled (pilose) trichomes; anthers 2–2.2 mm long, consisting of 1.3–1.5 × 0.9 × 0.5 (thick) mm thecae, 0.5 mm basal saccate appendage, and 0.1 mm acute connective tip, basifixed, free (not laterally coherent along adjacent edges); thecae co-lateral with longitudinal dehiscence slits facing inner side of stamen, hirsute with stiff simple trichomes; basal appendage facing inner (dehiscence) side of stamen, glabrous; connective, tip, and basal appendage darkened and appearing glandular. *Staminodes* 3, free, 11–11.5 mm long; filament portion 9–9.5 mm, flattened and resembling slightly reduced fertile anthers, 0.2 mm wide at base to 0.5 mm distally, distal part pilose; apical tip extension 1.8–2 × 0.2 mm, subulate, undifferentiated with no trace of abortive thecae or distinction between filament apex and connective, glabrous to sparsely hirsute. *Ovary* 0.5 mm (high) × ca 1 mm (wide), 5-lobed (apocarpous), glabrous, surrounded by a thin cupular disc; disc erect, distinctly shorter than ovary, 0.3 mm high, slightly lobed at apex. *Style* single, 6 × 0.1 mm, glabrous; stigma ca 0.3 mm long, obliquely 5-lobed, smooth. Fruit not seen.

**Figure 1.**
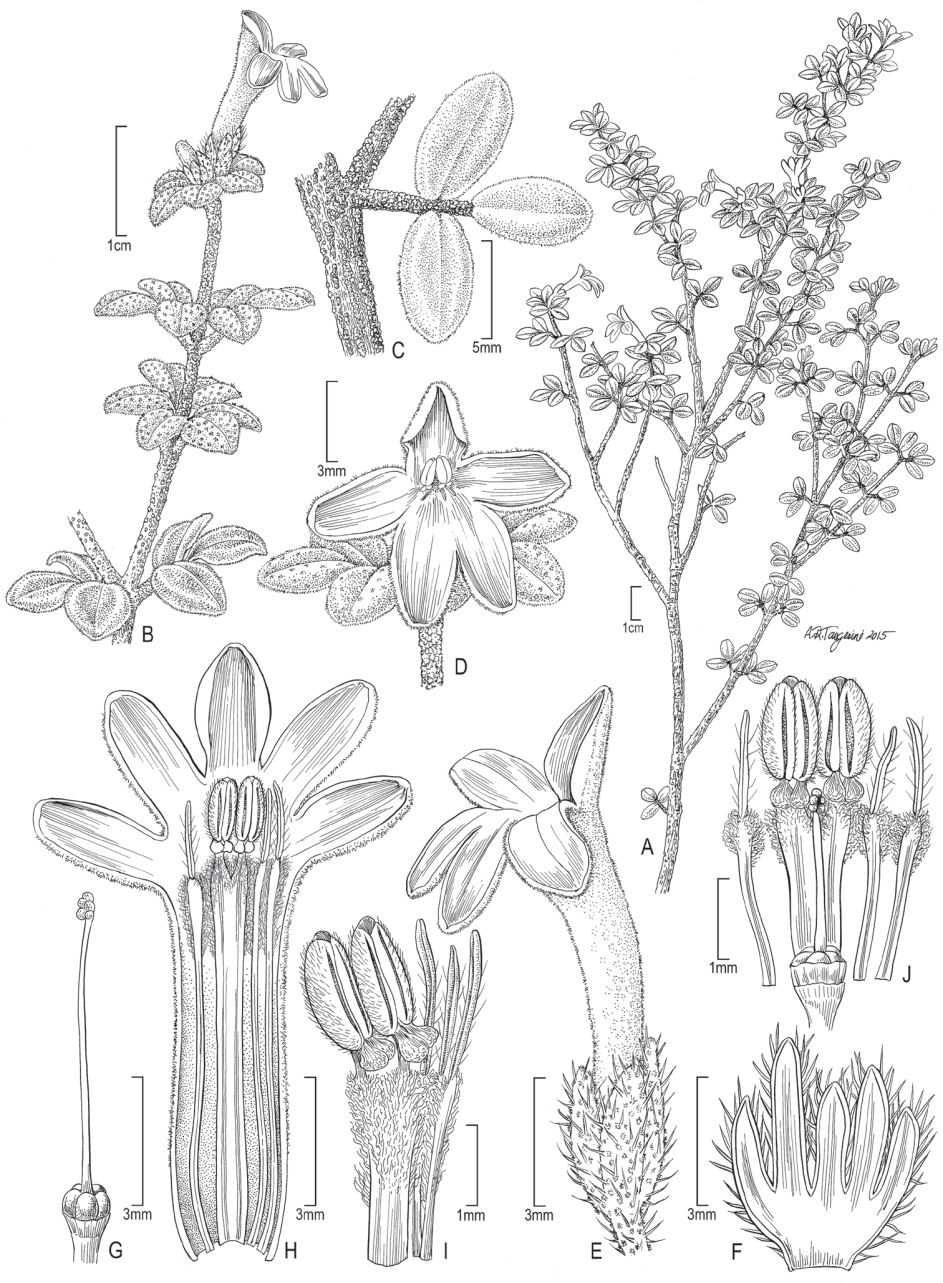
Illustration of *Raveniopsis microphyllus*. **A** Habit **B** Flowering branch **C** Trifoliate leaf **D** Flower (face view) **E** Flower (lateral view) **F** Calyx with five unequal sepals **G** Gynoecium **H** Corolla (split) and androecium **I** Androecium of bud **J** Floral dissection of same bud. (Source: *Tripp 3191*, US).

#### Etymology.

The specific epithet is derived from *micro*- (Greek, little or small) and -*phyllus* (Greek, -leaved), and refers to the small leaflet size, which is more reduced than in any other species of *Raveniopsis*.

#### Distribution and ecology.

*Raveniopsis microphyllus* is known only from the summit of Kamakusa Mtn. where it was occasional along a transect cut across the north-south axis of the narrow summit (personal observation). Flowers and young buds, although infrequent, were collected in June. The cold, wet, windswept summit of Kamakusa Mtn. is covered by a relatively low-stature (2–4 m tall) evergreen shrubland on peat overlying sandstone and can be classified in the upper montane life zone (Huber 1995b). The flora contains dense stands of *Bonnetia tepuiensis* Kobuski & Steyerm. and *B. roraimae* Oliv. (Bonnetiaceae), along with other montane elements such and *Schefflera monosperma* Maguire, Steyerm. & Frodin (Araliaceae), species of *Weinmannia* L. (Cunoniaceae), and at least one other undescribed endemic plant (*Tryssophyton* Wurdack, Melastomatacaeae). Immediately south of Kamakusa Mtn. along the wet edge of the Pakaraima Mtns. continues an unexplored and poorly mapped montane ridgeline with peaks. This local region includes some other >1500 m elevations, that while lower than the Kamakusa Mtn. summit (1686 m) are still within the 1500–3000 m highlands physiography (Huber 1995a). However, these nearby peaks appear less likely to contain additional populations of *R. microphyllus* due to their small sizes, and lower elevations that tend to support slightly different and taller plant communities. The closest peak (55 km SSE) to reach or exceed the elevation of Kamakusa Mtn. is relatively well-explored Mt. Ayanganna (2041 m), which has so far only yielded *Raveniopsis ruellioides* (Oliv.) R.S. Cowan.

#### Conservation status.

Following the criteria and categories of IUCN (2012), *Raveniopsis microphyllus* is given a preliminary status of Vulnerable (VU D2) due to population very small or restricted (area of occupancy <20km^2^ and number of locations <5). The species has extremely limited suitable montane habitat and coupled with relatively small known population size is vulnerable to climate and land use changes.

The upper part of Kamakusa Mtn. is presently pristine and undisturbed habitat, and the sole known *Raveniopsis microphyllus* population had no evidence of being unhealthy or fluctuating. The region is at risk of habitat destruction due to placer gold mining, although such activity is unlikely to reach the small, inhospitable summit of Kamakusa Mtn. Recent gold mining of moderate scale has occurred along the upper Partang River on the southern edge of Kamakusa Mtn. and its drainage, about 10 km from the type locality. While those mining operations had ceased by 2012, other waves of gold prospecting activities go back decades and have pushed further in. Such activities were noted as “pork-knocker camps” on Tillett et al. herbarium labels of 1960 (e.g., *Aechmea pallida* L.B. Sm. [Bromeliaceae], *Tillett 44859*, NY), and during the recent fieldwork (personal observation) that encountered old camps (furthest human intrusion was a long overgrown camp at N05°51'44.4", W060°09'20.5", 1019 m, and 6.2 km from the type locality), an abandoned unpaved runway (Partang airstrip), and ATV trails from the nearest village (Imbaimadai), which were constructed to support the recent upper Partang River mining operation.

## Discussion

In species keys (i.e., Cowan 1960; Steyermark 1980; Kallunki 2005) *Raveniopsis microphyllus* would quickly group with the three other compound-leaved species, *R. cowaniana* Steyerm. & Luteyn, *R. stelligera* (R.S. Cowan) R.S. Cowan (including *R. liesneri* Steyerm.), and *R. trifoliata* R.S. Cowan. However, those taxa are very different from *R. microphyllus* in much larger leaflet size, different types of trichomes, red-orange tubular corollas, and biogeography. They occur in Venezuela at different elevations on Sierra de la Neblina where *R. cowaniana* and *R. trifoliata* are endemic, and slightly broader-ranged *R. stelligera* also extends to Cerro Duida and Cerro Yutajé. Compound leaves aside, *Raveniopsis microphyllus* appears most morphologically similar to *R. breweri* Steyerm. in small, ovate lamina, white flowers, and trichome type (see below). *Raveniopsis microphyllus* has trifoliate leaves with very small leaflets (5–8.8 × 3–5.3 mm) that are adaxially pubescent when young (becoming glabrescent), a unique form of small rosulate trichomes, single (to few) flowered subsessile inflorescences, small sepals (largest 3 × 1 mm), small corollas, and hirsute anthers. In contrast, *R. breweri* has larger (22–32 × 10–16 mm), simple leaves that are adaxially glabrous except along the midvein, larger flattened rosulate trichomes, pedunculate 3–7 flowered racemose inflorescences, larger sepals (largest 8 × 2 mm), larger corollas, and glabrous anthers. In *R. breweri* the suggestion (fig. 1 in Steyermark 1980) that two staminodes are laterally conjoined to the stamens was not confirmed here, although the type collection was not examined. My observations of *R. breweri* (*Huber & Medina 8529*, US; *Prance & Huber 28254*, US) are that all three staminodes are free, although distally loosely confluent due to tangled trichomes. In addition, the two anthers are laterally coherent as has been found in some species of *Raveniopsis* and other Angostura Alliance genera (personal observation; Kallunki 1991; El Ottra et al. 2013), but which does not appear to be the case in *R. microphyllus* where the anthers are free in bud and at dehiscence. *Raveniopsis ruellioides*, the only other species in Guyana, differs in larger, simple, pellucid-punctate leaves with exclusively simple trichomes, and long pedunculate inflorescences with red flowers and foliaceous sepals.

**Figure 2.**
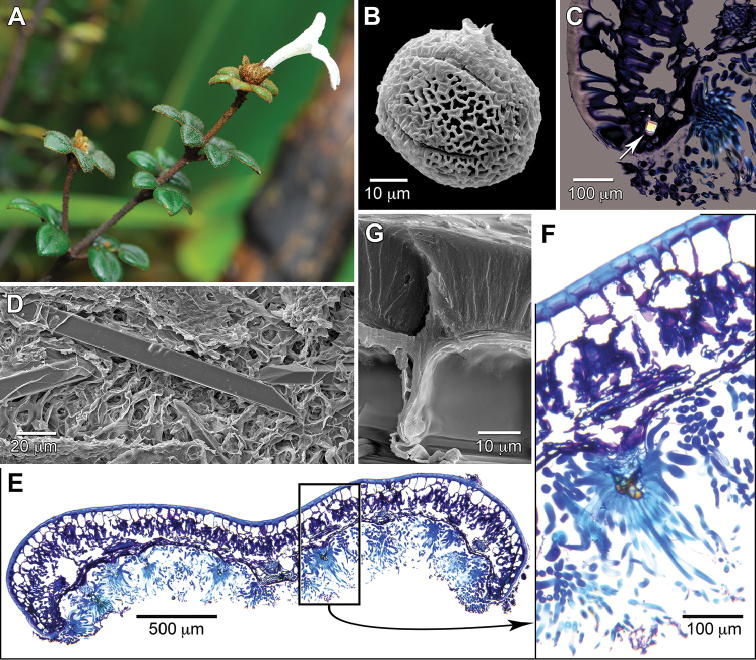
Morphology and anatomy of *Raveniopsis microphyllus*. **A** Life photo of flowering branch showing strongly zygomorphic, white corolla **B** Pollen with mesocolpium-centered equatorial view of whole grain **C** Transverse section of leaflet edge under polarized light, showing bright styloid crystal (at arrow) **D** In-situ styloid crystals near secondary vein **E** Transverse section of entire leaflet (tiled image) **F** Transverse section in closer view showing thick adaxial cuticle, and abaxial trichome with multicelled stalk **G** Adaxial epidermis showing thick cuticle above epidermal cells. (Source: *Tripp 3191*, US).

**Figure 3.**
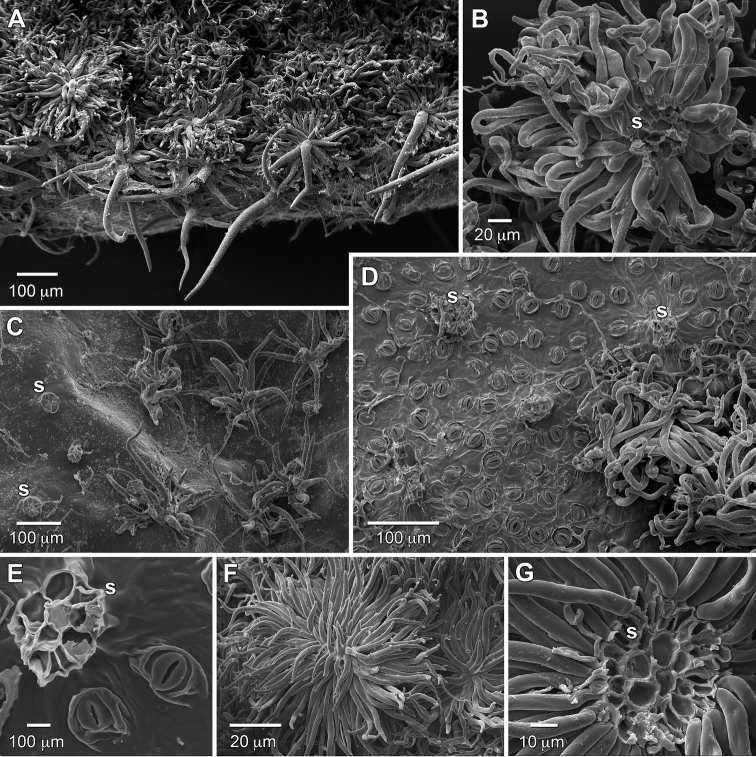
Trichome and leaf surface comparisons of *Raveniopsis microphyllus* (**A–E**) and *R. breweri* (**F–G**). **A** Abaxial side of leaflet showing trichomes transitioning from rosulate to porrect-multiradiate with central long radii at margin (sampled from proximal margin of lateral leaflet) **B** Back of rosulate trichome showing central multicellular attachment stalk, and curled free arms **C** Adaxial side of young leaflet showing multiradiate trichomes, and rosette bases of trichomes that have weathered off **D** Abaxial side of leaflet with most trichomes removed to show dense stomata and stalk bases of rosulate trichomes; note fungal hyphae on epidermis **E** Close-up of stomata and stalk base of rosulate trichome **F** *Raveniopsis breweri* abaxial trichome **G** Back of *Raveniopsis breweri* abaxial trichome, showing central multicellular attachment stalk. (Abbreviations: s = stalk. Source: *R. microphyllus*, **A–E** *Tripp 3191*, US; *R. breweri*, **F–G** *Prance & Huber 28254*, US).

The leaves of *Raveniopsis microphyllus* appear well adapted for a montane environment due to their small size, considerable vestiture, thick cuticle, and coriaceous nature. Pellucid dots (oil-containing secretory cavities), which are a characteristic feature of Rutaceae leaves, are not visible in *R. microphyllus*, nor do they appear differentiated anatomically. They are surficially visible in some thinner-leaved species (e.g., *R. necopinata* Kallunki, *R. ruellioides*, and *R. stelligera*) but are not similarly obvious in other coriaceous species. I also did not note them elsewhere in *R. microphyllus* (i.e., floral parts during wet dissections), although some darkened surficial cells on the anthers may be secretory cells. Unfortunately at the time of collection no attention was paid to any presence of citrus or resinous odor that would indicate essential oils. Leaf anatomy (Fig. 2E, F) shows a dorsiventral structure and a thick cuticle (Fig. 2G; 20–23 μm at the thinnest point mid-cell and thicker where adjacent cells meet, as measured via SEM) on the large-celled epidermis of the glabrescent adaxial side. The epidermis of the abaxial side is small-celled and lacks the cuticle thickening. Stomata are confined to the abaxial side where they are densely packed under the canopy of stalked trichomes (Fig. 3D) and absent along the slightly raised secondary vein courses. The mesophyll is poorly differentiated into palisade and spongy layers. Elongate styloid crystals are sparsely present in the mesophyll and mostly oriented parallel to major veins. The crystals are square in transverse section (Fig. 2C) and have obliquely faceted, needle-like ends (Fig. 2D).

The indument on *Raveniopsis* is very diverse in form (i.e., variations on glandular, simple, dendritic, multiradiate, and rosulate) both among species and often within an individual plant in a location-dependent manner. The interspecific trichome diversity is taxonomically useful (e.g., applied in the species key of Steyermark (1980)) and may be phylogentically informative, although care is needed to observe homologous plant structures when making fine comparisons. *Raveniopsis microphyllus* bears five different location-specific trichome forms, with some minor intergradation, that can be grouped into three basic types including: (1) simple, (2) rosulate, and (3) multiradiate. While rosulate can be considered a subtype of multiradiate (see Webster et al. 1996), the distinction is useful here for descriptive purposes. The simple trichomes are confined to inside the flower, and can be of two forms as either stiff and straight (strigose) on the anthers, or crinkled (pilose) on the filaments and adjacent inner corolla wall. The rosulate trichomes (Fig. 3A–B, D) are tufts with numerous soft, often twisted radii (arms) and are well developed on the abaxial laminar surface and along stems and petioles. The multiradiate trichomes (Fig. 3A, C) while similar to the rosulate form differ in having fewer and stiffer radii and are often further differentiated into a modified form with an erect longer central radius (porrect-multiradiate); they occur on the adaxial side and margins of the lamina, and outer surfaces of the flower. Both rosulate and multiradiate trichomes are stipitate with a short, fragile (allowing the trichomes to easily detach), non-vascularized multicellular stalk, which in cross section is a rosette of 6–9 cells surrounding a central cell (Fig. 3E). The trichomes and their stalks have differential staining relative to the site of epidermal attachment (Fig. 2F). After the trichomes weather off the adaxial side, the remnants of the stalks are visible as rosette outlines (Fig. 3C). The species of *Raveniopsis* most closely approaching *R. microphyllus* in laminar trichome morphology are *R. breweri*, *R. peduncularis* Pittier & Lasser (but not “appearing lepidote”, fide Kallunki 2005), and *R. jauaensis* Steyerm., that were grouped as “stellate-squamose” in the species key of Steyermark (1980). They share similar tufted rosulate appearance with *R. microphyllus* but are larger and tend to be flattened (i.e., squamose), due to loss or shortening of central radii (Fig. 2F, G). These differences are more pronounced in *R. peduncularis* and *R. jauaensis*, which are further distinguished from *R. breweri* in bearing red flowers on elongate dischasial inflorescences.

The reticulate pollen of *Raveniopsis* has been shown to have aperture (3, 5, 6- aperturate) and size variation (Morton and Kallunki 1993). Although I had little pollen of *R. microphyllus* available, it appears reticulate, 5-colporate, prolate-spheroidal, and 45–50 × 43–48 μm in size (polar:equatorial ratio 1.05; n = 3, via SEM) (Fig. 2B). The pollen of *R. microphyllus* is very similar to other species in details of its reticulate sculpture, and falls in between the two reported size classes (Morton and Kallunki 1993).

The historical biogeography of *Raveniopsis* is likely complex and interesting given the variety of distributional patterns represented by species that are widespread, isolated endemics, sympatric endemics, or Amazonian disjuncts. Moreover, the sympatric species are morphologically diverse and (mostly) not obvious local species complexes. *Raveniopsis microphyllus* is the easternmost member of the 16 tepui species, and separated by 230–250 km from potential Venezuelan close relatives on Auyán-tepui (3 spp.) and Macizo del Chimantá (2 spp.). *Raveniopsis breweri*, which is the most morphologically similar species to *R. microphyllus*, is among the Auyán-tepui endemics. *Raveniopsis ruellioides* is a variable species (especially in leaf shape) that is broadly distributed across the Guiana Shield in southern Venezuela and eastward into the Pakaraima Mtns. It tolerates a broad elevation range (300–2600 m), although is mostly montane (1500+ m) and usually reaches lower elevations along watercourses. In the Pakaraima Mtns. it geographically most closely approaches *R. microphyllus* on Mt. Ayanganna, but as previously noted is morphologically very different and likely distantly related. *Raveniopsis sericea* R.S. Cowan was reported from Guyana (Kallunki 2005; Funk et al. 2007) but this record appears to be incorrect as further documentation could not be found (MO, NY, US, Biological Diversity of the Guiana Shield specimen database; J. Kallunki, personal communication). Otherwise that high altitude (1800–2500 m) species is endemic to the Macizo del Chimantá in Bolívar, Venezuela.

## Supplementary Material

XML Treatment for Raveniopsis microphyllus

## References

[B1] Bruniera CP Kallunki JA Groppo M (2015) *Almeidea* A. St.-Hil. belongs to *Conchocarpus* J.C. Mikan (Galipeinae, Rutaceae): evidence from morphological and molecular data, with a first analysis of subtribe Galipeinae PloS ONE 10(5): e0125650. https://doi.org/10.1371/journal.pone.0125650 10.1371/journal.pone.0125650 PMC4423776 25951371

[B2] Cowan RS (1960) Rutaceae. In: Maguire B Wurdack JJ and Collaborators The botany of the Guyana Highland––Part IV. Memoirs of the New York Botanical Garden 10: 24–34.

[B3] El Ottra JHL Pirani JR Endress PK (2013) Fusion within and between whorls of floral organs in Galipeinae (Rutaceae): structural features and evolutionary implications. Annals of Botany 111: 821–837. https://doi.org/10.1093/aob/mct039 23463590 10.1093/aob/mct039 PMC3631327

[B4] Funk V Hollowell T Berry P Kelloff C Alexander SN (2007) Checklist of the plants of the Guiana Shield (Venezuela: Amazonas, Bolivar, Delta Amacuro; Guyana, Surinam, French Guiana). Contributions from the United States National Herbarium 55: 1–584.

[B5] Groppo M Pirani JR Salatino ML Blanco SR Kallunki JA (2008) Phylogeny of Rutaceae based on two noncoding regions from cpDNA. American Journal of Botany 95: 985–1005. https://doi.org/10.3732/ajb.2007313 21632420 10.3732/ajb.2007313

[B6] Groppo M Kallunki JA Pirani JR Antonelli A (2012) Chilean *Pitavia* more closely related to Oceania and Old World Rutaceae than to Neotropical groups: evidence from two cpDNA non-coding regions, with a new subfamilial classification of the family. PhytoKeys 19: 9–29. https://doi.org/10.3897/phytokeys.19.3912 10.3897/phytokeys.19.3912 PMC3597001 23717188

[B7] Huber O (1995a) Geographical and physical features. In: Berry PE Holst BK Yatskievych K (Eds) Flora of the Venezuelan Guyana. Volume 1: Introduction. Missouri Botanical Garden Press, St. Louis, 1–61.

[B8] Huber O (1995b) Vegetation. In: Berry PE Holst BK Yatskievych K (Eds) Flora of the Venezuelan Guyana. Volume 1: Introduction. Missouri Botanical Garden Press, St. Louis, 97–160.

[B9] IUCN (2012) IUCN Red List Categories and Criteria: Version 3.1. Second edition. IUCN, Gland & Cambridge, 32 pp.

[B10] Kallunki JA (1991) Two new species of *Raveniopsis* (Rutaceae) from Amazonian Brazil. Boletim do Museu Paraense “Emilio Goeldi,” n.s. Botanica (Belem) 7: 301–307.

[B11] Kallunki JA (2005) Rutaceae. In: Berry PE Yatskievych K Holst BK (Eds) Flora of the Venezuelan Guyana. Volume 9 Rutaceae–Zygophyllaceae. Missouri Botanical Garden Press, St. Louis, 1–39.

[B12] Kubitzki K Kallunki JA Duretto M Wilson PG (2011) Rutaceae. In: Kubitzki K (Ed.) The families and genera of vascular plants, vol. 10: Flowering Plants: Eudicots (Sapindales, Cucurbitales, Myrtaceae). Berlin and Heidelberg, Germany, Springer Heidelberg, 276–356.

[B13] Morton CA Kallunki JA (1993) Pollen morphology of the subtribe Cuspariinae (Rutaceae). Brittonia 45: 286–314. https://doi.org/10.2307/2807604

[B14] Steyermark JA (1980) A new species of *Raveniopsis* (Rutaceae) from Venezuela. Brittonia 32: 47–50. https://doi.org/10.2307/2806217

[B15] Webster GL Del Arco Aguilar MJ Smith BA (1996) Systematic distribution of foliar trichome types in *Croton* (Euphorbiaceae). Botanical Journal of the Linnean Society 121: 41–57. https://doi.org/10.1111/j.1095-8339.1996.tb00744.x

[B16] Wurdack K Tripp E Radosavljevic A Redden K (2013) Kamakusa Expedition 2012: First botanical exploration of a remote Guyana tepui. The Plant Press 16(1), n.s.: 10–.$6 http://botany.si.edu/pubs/plantpress/vol16no1.pdf

